# Prognostic role of preoperative inflammatory markers in postoperative lung metastasis of colorectal cancer: a retrospective study

**DOI:** 10.1186/s12876-025-04091-8

**Published:** 2025-07-01

**Authors:** Jiukang Sun, Weili Xiong, Zhang Fang, Chuanjun Song

**Affiliations:** 1 Department of Oncology, The First Affiliated Hospital of Nanjing Medical University, Nanjing, Jiangsu 210029 China; 2 Department of Oncology, The Second People’s Hospital of Lianyungang & The Oncology Hospital of Lianyungang, Lianyungang, Jiangsu 222000 China; 3 Department of Cardiology, Taikang Xianlin Drum Tower Hospital, Affiliated Hospital of Medical School, Nanjing University, Nanjing, 210029 China; 4 Department of Oncology, Xinghua People’s Hospital Affiliated to Yangzhou University, No. 419 Yingwu South Road, Xinghua, Jiangsu 225700 China

**Keywords:** Colorectal cancer, Prognostic value, Inflammatory markers, Progression-free survival, Lung metastasis

## Abstract

**Background:**

This study aims to investigate the predictive significance of inflammatory markers for postoperative lung metastasis in colorectal cancer (CRC) patients. The focus is on exploring the relationship between traditional inflammatory markers, such as neutrophil-to-lymphocyte ratio (NLR), platelet-to-lymphocyte ratio (PLR), and newly introduced indices like C-reactive protein (CRP)–albumin–lymphocyte (CALLY), and their association with CRC progression and lung metastasis.

**Methods:**

A retrospective review of medical records from 303 CRC patients at the First Affiliated Hospital of Nanjing Medical University (January 2014 to December 2023) was conducted. Various inflammatory markers, baseline characteristics, and survival data were analyzed. Statistical analyses, including univariate and multivariate Cox regression and logistic regression, were performed to determine associations between inflammatory factors and CRC outcomes.

**Results:**

Among the patients, 55 developed lung metastasis during the study period. The results revealed that PNI (HR 0.368, 95% CI 0.235–0.577; *p* < 0.001, Table 2), LMR (HR 0.383, 95% CI 0.223–0.657; *p* < 0.001, Table 2), and CALLY (HR 0.18, 95% CI 0.111–0.294; *p* < 0.001) were associated with progression free survival (PFS). Moreover, PNI (HR 0.252, 95% CI 0.137–0.461; *p* < 0.001, Table 3) and CALLY (HR 0.11, 95% CI 0.0.05–0.245; *p* < 0.001, Table 3) emerged as independent risk factors for postoperative lung metastasis. And PNI (*p* = 0.028, Table 5) is more specific for predicting pulmonary metastasis in CRC.

**Conclusion:**

This study underscores the importance of inflammatory markers in predicting postoperative outcomes for CRC patients. Lower PNI, LMR, and CALLY were identified as significant predictors of reduced progression free survival, while PNI and CALLY were independently associated with an increased risk of postoperative lung metastasis. Further analysis demonstrated that PNI is a specific indicator for predicting the occurrence of pulmonary metastasis in CRC, independent of metastases to other sites. These findings highlight the potential clinical utility of these inflammatory markers in monitoring CRC recurrence and metastasis.

**Trial registration:**

The study was conducted according to the guidelines of the Declaration of Helsinki, and approved by the Ethics Committee of the First Affiliated Hospital of Nanjing Medical University (protocol code 2024—SR-066, date of approval 2024-02-27).

## Introduction

Colorectal cancer (CRC) is a leading cause of cancer-related deaths [1]. Current treatment modalities for CRC include surgery and chemotherapy, achieving a 90% 5-year survival rate for patients in various stages. Despite advances in treatment, distant metastasis remains a critical determinant of survival in colorectal cancer patients. Once distant metastasis occurs, the prognosis remains poor [2]. Postoperative metastasis is a major cause of death in CRC patients and is closely associated with tumor resistance [3]. The liver is the most common site of CRC metastasis, followed by the lungs [4]. We chose to focus on lung metastasis due to the differences in prognosis and mechanisms of occurrence. Previous studies have demonstrated that several different molecules are involved in the development of CRC metastasis. For example, chemokine receptors such as CXC motif chemokine ligand (CXCL) 12 and CXC chemokine receptor (CXCR) 4 have been implicated in liver metastasis and tumor recurrence in CRC patients [5]. In contrast, the activation of CXCR7 has been shown to promote lung metastasis in CRC [6]. Furthermore, the activation of the Wnt signaling pathway and the expression of ERBB2 interacting protein-positive B cells have been found to be more strongly associated with CRC lung metastasis [7, 8]. Studies have indicated that patients with lung metastasis generally have a longer survival time, potentially due to a more active immune microenvironment in the lungs and limited tumor cell growth within lung tissue [9]. Unlike liver metastasis, the mechanisms of lung metastasis remain incompletely understood.

A substantial body of research confirms that systemic inflammation is an integral component of the tumor immune microenvironment and plays a crucial role in the initiation, progression, and metastasis of tumors [10, 11]. Studies indicate a correlation between chronic inflammation and over 15% of human cancers [12]. Uncontrolled inflammation can promote tumor advancement through mechanisms such as DNA damage and reduced genomic stability [13]. Several traditional inflammatory markers, including neutrophil-to-lymphocyte ratio (NLR), platelet-to-lymphocyte ratio (PLR), systemic immune-inflammation index (SIRI), lymphocyte-to-monocyte ratio (LMR), and prognostic nutritional index (PNI), have been demonstrated to play an important role in the tumor immune microenvironment, particularly in tumor progression, metastasis, and response to chemotherapy [11, 14–16].

The C-reactive protein (CRP)–albumin–lymphocyte (CALLY) index, developed by Müller et al. in 2021, represents a novel inflammatory marker that integrates both the PNI and the CRP level [17]. The CALLY index has been demonstrated to be associated with the prognosis of liver cancer and CRC, with higher CALLY index values correlating with longer survival in patients [17, 18].

These markers are a combination of neutrophils, monocytes, platelets, albumin, and other indicators, reflecting the body’s inflammatory and nutritional status. Neutrophils regulate the tumor microenvironment by producing cytokines, chemokines, and growth factors, which can promote angiogenesis, tumor cell proliferation, and tumor cell migration [19]. Tumor-associated macrophages (TAMs), present in the tumor microenvironment, promote metastasis and immune suppression and are closely associated with peripheral blood monocyte counts [20]. Platelets stimulate tumor cell proliferation and promote angiogenesis by secreting a variety of cytokines and growth factors [21]. Malnutrition, on the other hand, leads to immune suppression, which weakens antitumor responses [22]. Therefore, these markers may also represent potential mechanisms that promote lung metastasis CRC.

However, the role of these inflammatory markers in CRC lung metastasis has not been studied, and thus, the main objective of our study is to fill this gap by exploring whether these markers can serve as a novel predictor to help clinicians better identify high-risk patients for lung metastasis.

## Materials and methods

### Study population

We retrospectively reviewed the medical records of patients diagnosed with primary CRC at the First Affiliated Hospital of Nanjing Medical University from January 2014 to December 2023. Inclusion criteria:

1. Age > 18 years;
2. Histological or cytological confirmation of primary CRC;
3. Patients underwent electively curative surgery (Coloctomy with enbloc removal of regional lymph nodes) for CRC;
4. Sufficient clinical and hematological data can be obtained.

Exclusion criteria:

1. With distant metastasis at the time of diagnosis;
2. With acute inflammation at the time of diagnosis;
3. History of concomitant inflammatory bowel disease;
4. History of receipt of neoadjuvant treatment;
5. Presence of other malignancies or concurrent chronic inflammatory conditions;
6. Patients with missing key data (including neutrophils, lymphocytes, monocytes, albumin, and CRP).

For each eligible patient, we collected the following information:

1. Basic patient information, including gender, age, tumor pathology, smoking history, alcohol consumption history, etc.;
2. Peripheral blood indices within the week before surgery, including routine blood tests, biochemistry test and the CRP level;
3. Postoperative chemotherapy (all patients with the history of adjuvant chemotherapy received standard CapeOx treatment);
4. Survival time. Namely PFS, the time from the initiation of operation to radiological or clinical progression or death from any cause.

The follow-up include:

1. Physical examination with an emphasis on digital rectal examination;
2. Blood carcino-embryonic antigen (CEA) levels;
3. Chest, abdominal, and pelvic CT;
4. Colonoscopy examination.

All patients were followed up on an ongoing basis, once every 3 months for 3 years, followed by once every 6 months to 5 years, and then once a year. The diagnosis of metastasis was independently confirmed by two radiologists through CT scans. Patients who had no progression or death at the last follow-up were considered censored.

This study obtained approval from the Ethics Committee of the First Affiliated Hospital of Nanjing Medical University and adheres to the principles of the Helsinki Declaration.

### Formulas

The formulas for the relevant indices in this study are as follows: $\:NLR=N/L$; $\:PLR=P/L$; $\:LMR=L/M$; $\:PNI=A\left(g/L\right)+5\times\:L(\times\:{10}^{9}/L)$; $\:\text{C}\text{A}\text{L}\text{L}\text{Y}\:=\:A\:(g/L)\:\times\:\:L(\times\:{10}^{9}/L)\:/\:\left(C\right(mg/L)\times\:10)$; $\:SIRI=N\times\:M/L$; $\:\:BMI={Weight\:\left(kg\right)/Height\:\left(m\right)}^{2}$. Here, N, L, P, and M represent neutrophil, lymphocyte, platelet, and monocyte counts, respectively. A and C represent albumin and C-reactive protein. The optimal cutoff values were calculated using the Youden index (Youden’s Index=Sensitivity+Specificity−1. This method ensures that the selected cutoff value strikes a balance between high sensitivity and specificity), resulting in the following cutoff values: NLR, 1.78; PLR, 157.79; PNI, 52.375; LMR, 2.08; CALLY, 2.29; SIRI, 0.87.

### Statistical analysis

Data analysis was conducted using IBM SPSS version 27.0. Kaplan-Meier curves were employed to analyze PFS. Non-critical missing data were imputed using multiple imputation methods. All variables underwent univariate Cox regression analysis, and only those with a significance level of *P* < 0.05 were included in the multivariate Cox regression analysis. Logistics regression analysis were used to explore the associations between inflammatory factors and baseline characteristic. A two-tailed P-value < 0.05 was considered statistically significant.

## Result

### Baseline characteristic

This study included a total of 303 patients with an average age of 62 years. Among them, 175 (57.8%) were male, and 128 (42.2%) were female. The mean follow-up duration was 22.2 months. A total of 124 patients eventually experienced disease progression due to metastasis. Among them, 55 patients (44.3%) developed lung-only metastases, 53 patients (42.7%) had liver-only metastases, 4 patients (3.2%) presented with ovary-only metastases, and 12 patients (9.7%) exhibited multiple-site metastases. The specific baseline characteristics and inflammatory markers of the patients are presented in Table 1.

**Table 1:** Baseline clinicopathological characteristics of patients

Characteristics		Number (%) (*n* = 303)
Age(years)	Median	62
	< 70	213(70.3)
	> 70	90(29.7)
Gender	Male	175(57.8)
	Female	128(42.2)
BMI (kg/m2)	< 18.5	34(11.2)
	18.5–24	226(74.6)
	> 24	43(14.2)
Tumor location	Right	118(38.7)
	Left	185(61.3)
Smoking status	Yes	33(10.9
	No	270(89.1)
Drinking status	Yes	19(6.3)
	No	284(93.7)
Hypertension	Yes	94(31)
	No	209(69)
Diabetes	Yes	25(8.3)
	No	278(91.7)
Adjuvant chemotherapy	Yes	290(95.7)
	No	13(4.3)
TNM stage	II	107(35.3)
	III	196(64.7)
NLR	High	199(65.7)
	Low	104(34.3)
PLR	High	132(43.6)
	Low	171(56.4)
PNI	High	249(82.2)
	Low	54(17.8)
LMR	High	249(92.1)
	Low	24(7.9)
CALLY	High	138(45.5)
	Low	165(54.5)
SIRI	High	159(52.5)
	Low	144(47.5)

*Abbreviations:BMI* Body Mass Index, *NLR* Neutrophil–lymphocyte ratio, *PLR* Platelet–lymphocyte ratio, *PNI* Prognostic nutritional index, *LMR* Lymphocyte–monocyte ratio, *CALLY* CRP–albumin–lymphocyte index, *SIRI* Systemic immune-inflammation index

### Associations between inflammatory markers and PFS

We performed univariate Cox regression analysis to assess the relationship between baseline characteristics, inflammatory markers, and PFS in CRC patients. The results showed that age (*p* < 0.001), hypertension (*p* = 0.03), diabetes (*p* = 0.01), adjuvant chemotherapy (*p* < 0.001), TNM stage (*p* = 0.001), NLR (*p* < 0.001), PNI (*p* = 0.03), LMR (*p* < 0.001), CALLY (*p* < 0.001), and SIRI (*p* = 0.009) were significant factors influencing PFS (all *p* < 0.05, Table 2). These factors were then included in multivariate Cox regression analysis. The multivariate analysis identified that PFS was independently associated with several factors. These included adjuvant chemotherapy (HR 0.384, 95% CI 0.188–0.735; *p* = 0.009, Table 2), TNM stage (HR 2.14, 95% CI 1.387–3.301; *p* < 0.001, Table 2), PNI (HR 0.368, 95% CI 0.235–0.577; *p* < 0.001, Table 2), LMR (HR 0.383, 95% CI 0.223–0.657; *p* < 0.001, Table 2), and CALLY (HR 0.18, 95% CI 0.111–0.294; *p* < 0.001, Table 2).

**Table 2:** Associations between inflammatory markers and PFS

		Univariate analysis			Multivariate analysis		
Characteristics		HR	95% CI	*P*-value	HR	95% CI	*P*-value
Age (years)	> 70/<70	1.891	1.316–2.718	< 0.001	1.285	0.873–1.893	0.20
Gender	Female/Male	0.919	0.641–1.319	0.64			
BMI	> 24/18.5–24/<18.5	2.932	0.586–4.238	0.42			
Tumor location	Right/Left	0.824	0.576–1.181	0.29			
Smoking status	Yes/No	1.013	0.58–1.769	0.96			
Drinking status	Yes/No	0.958	0.467–1.966	0.90			
Hypertension	Yes/No	1.489	1.033–2.146	0.03	1.031	0.683–1.558	0.88
Diabetes	Yes/No	1.912	1.144–3.195	0.01	0.795	0.42–1.507	0.48
Adjuvant chemotherapy	Yes/No	0.335	0.188–0.599	< 0.001	0.384	0.188–0.785	0.009
TNM stage	II/III	1.959	1.307–2.934	0.001	2.14	1.387–3.301	< 0.001
NLR	High/Low	2.194	1.423–3.384	< 0.001	1.382	0.79–2.419	0.25
PLR	High/Low	1.318	0.924–1.879	0.12			
PNI	High/Low	0.647	0.43–0.972	0.03	0.368	0.235–0.577	< 0.001
LMR	High/Low	0.329	0.203–0.533	< 0.001	0.383	0.223–0.657	< 0.001
CALLY	High/Low	0.151	0.095–0.241	< 0.001	0.18	0.111–0.294	< 0.001
SIRI	High/Low	1.626	1.131–2.338	0.009	1.101	0.687–1.763	0.69

*Abbreviations:BMI* Body Mass Index, *NLR* Neutrophil–lymphocyte ratio, *PLR* Platelet–lymphocyte ratio, *PNI* Prognostic nutritional index, *LMR* Lymphocyte–monocyte ratio, *CALLY* CRP–albumin–lymphocyte index, *SIRI* Systemic immune-inflammation index

**Fig. 1:**
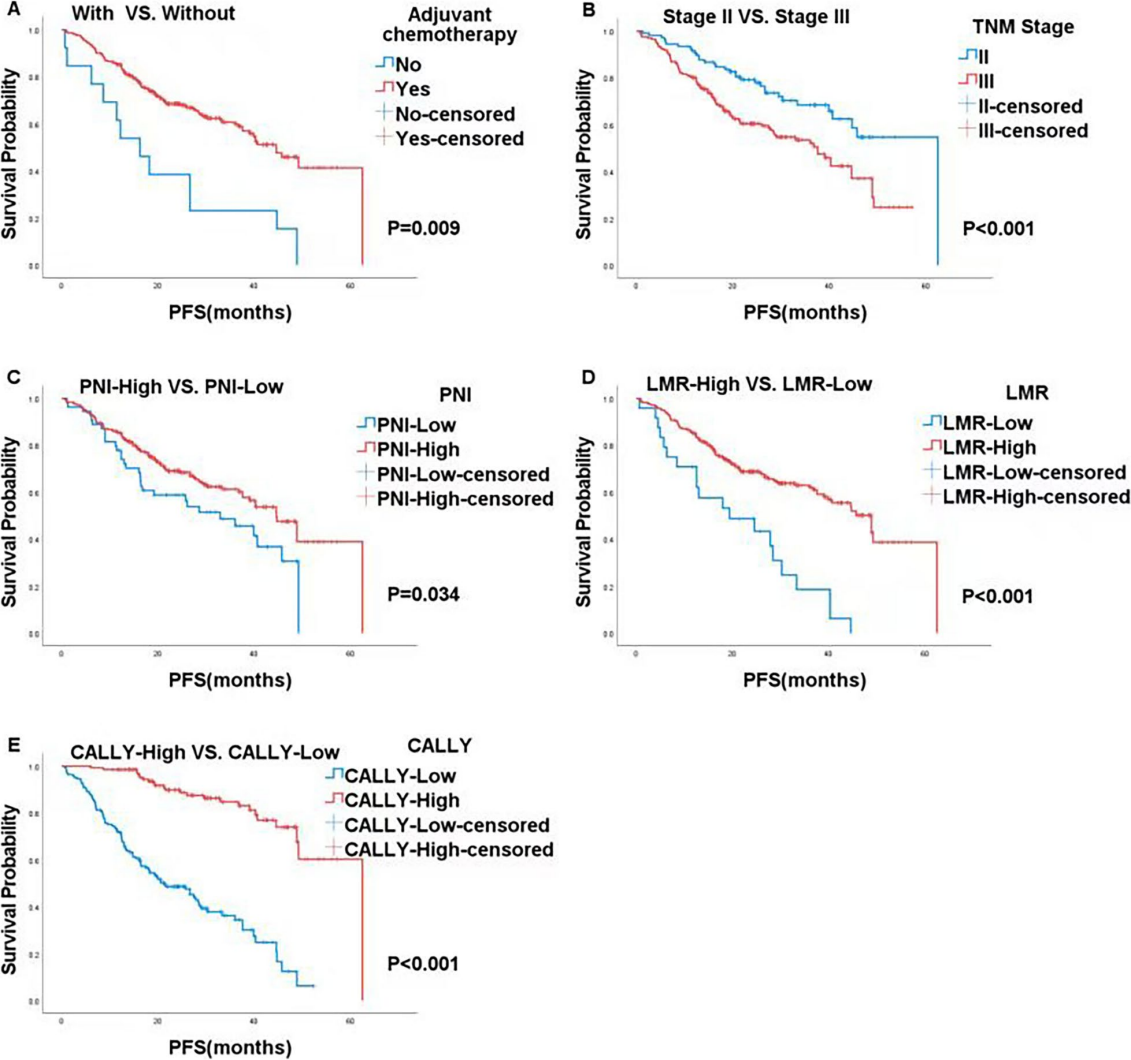
Kaplan-Meier analysis based on adjuvant chemotherapy, TNM stage, PNI, LMR and CALLY in all patients with CRC. **A** adjuvant chemotherapy; **B** TNM stage; **C** PNI; **D** LMR; **E** CALLY

Kaplan–Meier survival analysis further showed that patients who received adjuvant chemotherapy had a longer PFS (40.1 vs. 20.8 months, *P* = 0.009, Fig. 1). Patients with later TNM stages also had longer PFS (45.7 vs. 33.3 months, *P* < 0.001, Fig. 1). Moreover, patients with higher PNI (44.6 vs. 32.9 months, *P* = 0.034, Fig. 1), higher LMR (48.9 vs. 19.4 months, *P* < 0.001, Fig. 1), and higher CALLY (62.5 vs. 21.5 months, *P* < 0.001, Fig. 1) also experienced prolonged PFS.

Adjuvant chemotherapy and TNM stage were significant factors for PFS, highlighting the importance of post-surgical chemotherapy and early-stage detection. Lower PNI, LMR, and CALLY indicate poorer immune function and nutrition, leading to worse survival outcomes. This highlights the importance of considering inflammation and nutritional status in the prognosis of CRC patients.

### Associations between inflammatory factors and postoperative lung metastasis

Among the 303 patients, 55 developed lung metastasis by the last follow-up. In the univariate Cox regression analysis, inflammatory markers and baseline characteristics such as age (*p* = 0.03), hypertension (*p* = 0.01), TNM stage (*p* < 0.001), NLR (*p* = 0.03), PNI (*p* < 0.001), and CALLY (*p* < 0.001) were found to significantly impact the development of lung metastasis (all *p* < 0.05, Table 3). Subsequent multivariate Cox regression analysis showed that TNM stage (HR 3.455, 95% CI 1.654–7.22; *p* < 0.001, Table 3), PNI (HR 0.252, 95% CI 0.137–0.461; *p* < 0.001, Table 3), and CALLY (HR 0.11, 95% CI 0.05–0.245; *p* < 0.001, Table 3) were independent risk factors for postoperative lung metastasis in CRC patients.

**Table 3:** Associations between inflammatory factors and postoperative lung metastasis

		Univariate analysis			Multivariate analysis		
Characteristics		HR	95% CI	*P*-value	HR	95% CI	*P*-value
Age (years)	> 70/<70	1.824	1.051–3.163	0.03	1.435	0.814–2.531	0.21
Gender	Female/Male	1.005	0.585–1.727	0.98			
BMI	> 24/18.5–24/<18.5	0.945	0.823–1.215	0.84			
Tumor location	Right/Left	0.721	0.421–1.233	0.23			
Smoking status	Yes/No	1.813	0.911–3.609	0.09			
Drinking status	Yes/No	1.479	0.586–3.733	0.41			
Hypertension	Yes/No	1.983	1.154–3.407	0.01	1.18	0.659–2.113	0.57
Diabetes	Yes/No	1.941	0.914–4.123	0.08			
Adjuvant chemotherapy	Yes/No	0.438	0.172–1.113	0.08			
TNM stage	II/III	3.372	1.645–6.913	< 0.001	3.455	1.654–7.22	< 0.001
NLR	High/Low	1.994	1.049–3.789	0.03	1.658	0.834–3.297	0.14
PLR	High/Low	1.096	0.641–1.874	0.73			
PNI	High/Low	0.404	0.231–0.706	< 0.001	0.252	0.137–0.461	< 0.001
LMR	High/Low	0.493	0.21–1.16	0.11			
CALLY	High/Low	0.107	0.05–0.232	< 0.001	0.11	0.05–0.245	< 0.001
SIRI	High/Low	1.189	0.696–2.032	0.52			

*Abbreviations:BMI* Body Mass Index, *NLR* Neutrophil–lymphocyte ratio, *PLR* Platelet–lymphocyte ratio, *PNI* Prognostic nutritional index, *LMR* Lymphocyte–monocyte ratio, *CALLY* CRP–albumin–lymphocyte index, *SIRI* Systemic immune-inflammation index

**Fig. 2:**
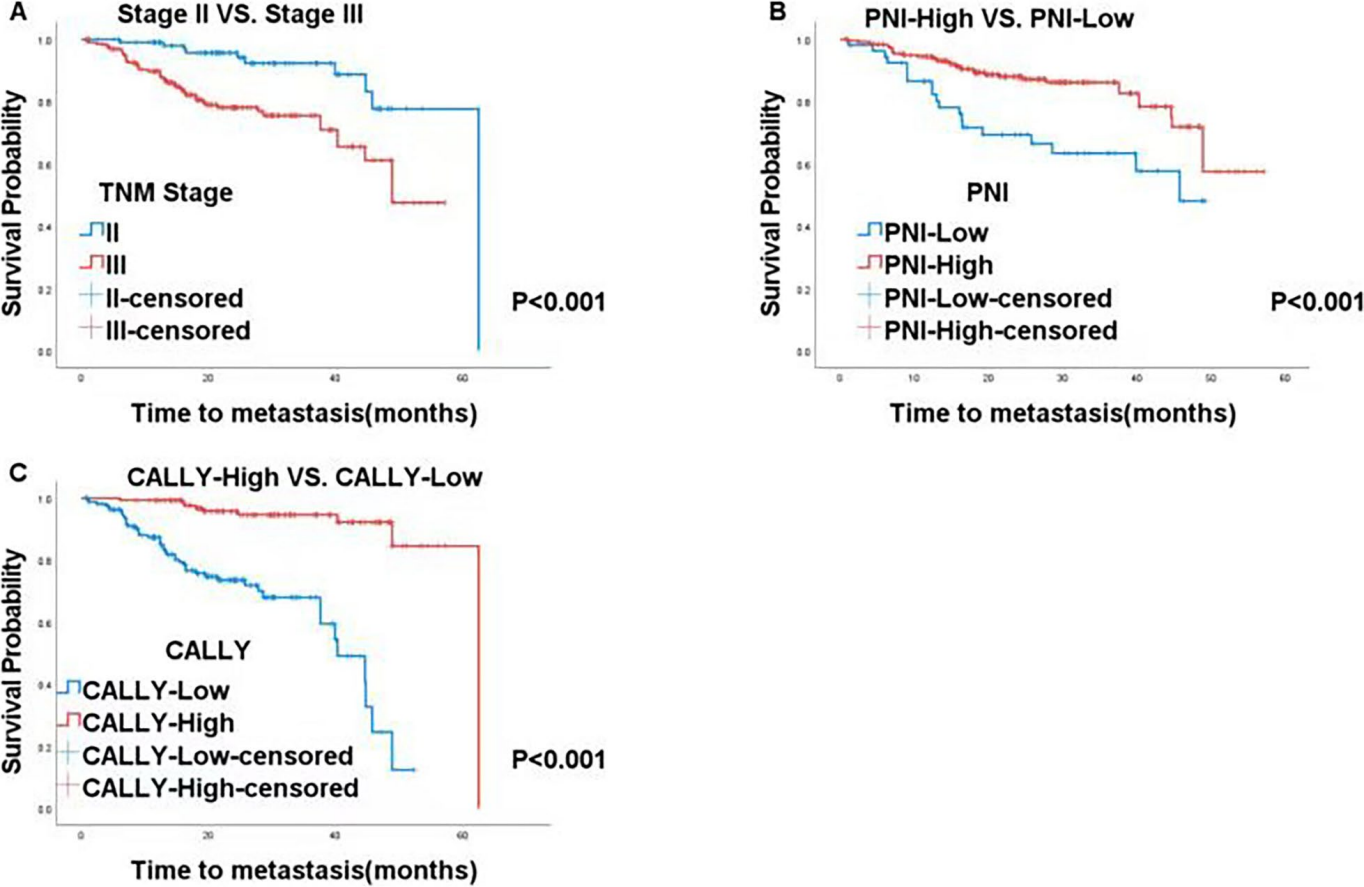
Kaplan-Meier analysis based on TNM stage, PNI and CALLY in all patients with CRC **A** TNM stage; **B** PNI; **C** CALLY

This was further supported by the Kaplan–Meier survival curves. Patients with later TNM stages (II vs. III, 52.3 vs. 46.9 months, *P* < 0.001, Fig. 2), low PNI (High vs. Low, 62.5 vs. 45.8 months, *P* < 0.001, Fig. 2), and low CALLY (High vs. Low, 62.5 vs. 40.3 months, *P* < 0.001, Fig. 2) were more likely to develop lung metastasis postoperatively. This suggests that PNI and CALLY could serve as indicators for postoperative lung metastasis in CRC patients, providing guidance for clinical treatment.

### Associations between inflammatory factors and baseline characteristic

We further explored the associations between PNI, LMR, CALLY, and baseline characteristics in CRC patients. The results indicated that CALLY was associated with age (*p* < 0.001), diabetes (*p* = 0.03) and TNM stage (*p* = 0.04) (all *p* < 0.05, Table 4), while there is no association between baseline characteristics and PNI and LMR (all *p* > 0.05, Table 4).

**Table 4:** Associations between inflammatory factors and baseline characteristic

Characteristics		*P*-value (PNI)	*P*-value (LMR)	*P*-value (CALLY)
Age, years	> 70/<70	0.15	0.32	< 0.001
Gender	Female/Male	0.71	0.95	0.27
BMI	> 24/18.5–24/<18.5	0.46	0.41	0.99
Tumor location	Right/Left	0.54	0.77	0.68
Smoking status	Yes/No	0.13	0.66	0.26
Drinking status	Yes/No	0.70	0.96	0.75
Hypertension	Yes/No	0.68	0.51	0.34
Diabetes	Yes/No	0.40	0.99	0.03
TNM stage	II/III	0.98	0.91	0.04

*Abbreviations:BMI* Body Mass Index, *PNI* Prognostic nutritional index, *LMR* Lymphocyte–monocyte ratio, *CALLY* CRP–albumin–lymphocyte index

### Specificity of inflammatory markers in predicting lung metastasis of CRC

To further evaluate whether PNI and CALLY possess specificity in predicting pulmonary metastasis in colorectal cancer, we compared these indicators between patients with lung metastases and those with metastases to other sites. The results showed a significant difference in PNI between the two groups (*p* = 0.028, Table 5), suggesting that PNI not only serves as a general prognostic indicator but may also provide insight into site-specific metastatic patterns, particularly pulmonary metastasis. In contrast, CALLY did not show a statistically significant difference between the groups (*p* = 0.456, Table 5), indicating that its value in predicting pulmonary metastasis may be relatively limited. Instead, CALLY may be more suitable for evaluating overall prognosis rather than distinguishing between specific metastatic sites.

**Table 5:** Comparison of lung metastases from colon cancer with metastases at other sites

Characteristics		Patients with Metastasis(*n* = 124, %)	Patients with Lung Metastasis(*n* = 55, %)	Patients with Other Metastasis(*n* = 69, %)	*P*-value
Age, years	> 70	75(60.5)	21(38.2)	54(78.3)	0.788
	< 70	49(39.5)	34(61.8)	15(21.7)	
Gender	Male	73(58.9)	31(56.4)	42(60.9)	0.616
	Female	51(41.1)	24(43.6)	27(39.1)	
BMI	Mean ± SD	23.30 ± 3.47	23.29 ± 3.50	23.32 ± 3.52	0.592
Tumor location	Right	51(41.1)	24(43.6)	27(39.1)	0.616
	Left	73(58.9)	31(56.4)	42(60.9)	
Smoking status	Yes	14(11.3)	10(18.2)	4(5.8)	0.030
	No	110(88.7)	45(81.8)	65(94.2)	
Drinking status	Yes	8(6.5)	5(9.1)	3(4.3)	0.289
	No	116(93.5)	50(90.9)	66(95.7)	
Hypertension	Yes	48(38.7)	25(45.5)	23(33.3)	0.171
	No	76(61.3)	30(54.5)	46(66.7)	
Diabetes	Yes	17(13.7)	8(14.6)	9(13.0)	0.811
	No	107(86.3)	47(85.4)	60(87.0)	
Adjuvant chemotherapy	Yes	111(89.5)	50(90.9)	61(88.4)	0.654
	No	13(10.5)	5(9.1)	8(11.6)	
NLR	High	97(78.2)	42(76.4)	55(79.7)	0.657
	Low	27(21.8)	13(23.6)	14(20.3)	
PLR	High	64(51.6)	26(47.3)	38(55.1)	0.392
	Low	60(48.4)	29(52.7)	31(44.9)	
PNI	High	93(75.0)	36(65.5)	57(82.6)	0.028
	Low	31(25.0)	19(34.5)	12(17.4)	
LMR	High	104(83.9)	49(89.1)	55(79.7)	0.161
	Low	20(16.1)	6(10.9)	14(20.3)	
CALLY	High	24(19.4)	9(16.4)	15(21.7)	0.456
	Low	100(80.6)	46(83.6)	54(78.3)	
SIRI	High	75(60.5)	29(52.7)	46(66.7)	0.119
	Low	49(39.5)	26(47.3)	23(33.3)	

*Abbreviations:BMI* Body Mass Index, *PNI* Prognostic nutritional index, *LMR* Lymphocyte–monocyte ratio, *CALLY* CRP–albumin–lymphocyte index

## Discussion

CRC is one of the most common malignancies worldwide, and metastasis is a major cause of postoperative mortality in CRC patients [3]. The liver is the most common site of metastasis in CRC patients, followed by lung metastasis [4]. Currently, research on the relationship between liver metastasis and inflammatory markers is well-established, but the association between lung metastasis and inflammatory markers remains unclear [23]. Therefore, this study explores the connection between inflammatory markers and postoperative PFS as well as postoperative lung metastasis in CRC patients.

Systemic inflammation is closely associated with the occurrence, development, and metastasis of tumors [10]. Inflammatory cells can release various cytokines and promote tumor progression and metastasis through oxidative stress and other mechanisms [24, 25]. Ulcerative colitis is a chronic inflammatory bowel disease, and patients with inflammatory bowel disease have an increased risk of developing colorectal cancer. Burr et al. found that non-steroidal anti-inflammatory drugs can reduce the risk of malignancy in inflammatory bowel disease, suggesting that inflammation plays an important role in the malignant transformation of inflammatory bowel disease [26]. Therefore, we will focus the discussion on the impact of inflammation and its related markers on cancer.

Neutrophils, lymphocytes, and monocytes are important components of the tumor microenvironment, playing crucial roles in tumor progression and metastasis [13].

Neutrophils can promote tumor occurrence by releasing reactive oxygen species or proteases and participate in tumor angiogenesis, thereby facilitating tumor progression [11]. Neutrophils can also inhibit the cytotoxic effects of lymphocytes on tumors by releasing various enzymes. When neutrophils are reduced or CXCR2 is blocked to inhibit their chemotaxis, tumor angiogenesis can be reduced [27]. As a traditional inflammatory marker, the predictive value of the NLR has long been demonstrated in various tumors, including CRC [11].

Lymphocytes are crucial components of the body’s defense against tumors, with CD8 + T cells directly attacking tumor cells and CD4 + T cells secreting cytokines and antibodies to activate NK cells and CD8 + T lymphocytes, thereby exerting their anti-tumor effects [28]. Higher infiltration of lymphocytes in the tumor microenvironment often signifies a better prognosis [29]. Conversely, reduced lymphocytes are considered adverse prognostic factors for cancer patients. Additionally, individuals with immune deficiencies are more susceptible to developing tumors, particularly those induced by infections [30]. During the process of carcinogenesis, there is often an increase in circulating neutrophils and a decrease in lymphocytes [23]. Huang et al. demonstrated that the percentage of lymphocytes before treatment serves as a reliable prognostic indicator for lung cancer, where a higher percentage of lymphocytes represents a better prognosis [15]. Multiple clinical studies have also suggested an association between elevated lymphocytes and improved tumor prognosis [16, 31, 32]. This aligns with our findings, indicating that CRC patients with higher PNI (indicating higher lymphocytes) experience longer PFS and are less prone to lung metastasis after surgery.

Monocytes play a crucial role in the process of pathogen clearance, but these cells can also simultaneously promote local inflammation [33]. Research indicates that monocytes can facilitate tumor growth and metastasis. In a mouse model of colorectal cancer, monocytes were recruited to primary tumors and lung metastases through chemokines, thereby promoting tumor migration and progression [34]. Katarzyna et al. found that CRC patients with low absolute monocyte counts before surgery had a longer 5-year disease-free survival [16]. This suggests that monocytes may promote tumor progression, supporting our conclusion that CRC patients with higher LMR (indicating lower monocytes) experience longer PFS after surgery.

CRP is one of the most commonly used indicators to assess systemic inflammation, and its levels are generally positively correlated with the degree of inflammation [35]. Inflammation induces various types of damage to nucleic acids through the production of reactive oxygen species (ROS), leading to tissue damage. This tissue damage can activate progenitor cells/stem cells for tissue regeneration. Stem cells are damaged by ROS/reactive nitrogen species (RNS) derived from inflammation, and the mutations generated can accumulate, potentially contributing to the mechanism of cancer stem cell formation [36]. Increasing evidence suggests that epigenetic silencing, through the downregulation of tumor suppressor genes and microRNAs, plays a crucial role in tumorigenesis [37]. In the inflammatory microenvironment, pro-inflammatory cytokines such as interleukin-6 (IL-6) and reactive oxygen species can transcriptionally influence DNA methyltransferase 1 (DNMT1) protein, leading to enhanced DNA methylation of tumor suppressor genes and microRNAs. This enhanced methylation results in the functional loss of tumor suppressor genes, thereby contributing to tumorigenesis [38]. Serum albumin is one of the key indicators reflecting the nutritional status of the body [39]. Nutritional status plays an important role in the development and progression of tumors, especially in gastrointestinal cancers. On one hand, CRC cells influence nutrient absorption and utilization through inflammatory and metabolic processes, making CRC patients prone to malnutrition [40, 41]. On the other hand, due to gastrointestinal symptoms affecting appetite and food intake, most CRC patients face varying degrees of malnutrition risk [40]. Malnutrition can lead to a decrease in lymphocyte count and lymphocyte dysfunction, suppressing immune function and thereby promoting tumor progression [42]. Studies have shown that high CRP in solid tumor patients is associated with increased recurrence, metastasis, and mortality rates in 90% of reports [14]. Nozoe et al. found that preoperative CRP levels could predict early recurrence in CRC, with patients exhibiting elevated preoperative CRP experiencing significantly higher rates of liver and lymph node metastasis and poorer prognosis, suggesting that CRP may serve as an effective prognostic indicator [43]. Yang et al. also demonstrated that lower CALLY is associated with an increased risk of death in CRC patients [18]. This aligns with our study, where lower CALLY (indicating higher CRP and poor nutrition) was significantly correlated with postoperative lung metastasis and poorer prognosis in CRC patients.

However, some traditional inflammatory markers, such as NLR, PLR, and LMR, were not found to be independently associated with lung metastasis or PFS in our study. These inflammatory markers, however, have been shown in previous studies to be associated with tumor PFS or distant metastasis. Potential reasons are as follows: (1) In our multivariate analysis, besides LMR, other inflammatory markers (such as PNI and CALLY) were also included. These variables may share some collinearity with each other. Because these markers potentially overlap in certain mechanisms, they might share some of the independent prognostic information, resulting in PLR and NLR not retaining statistical significance in the multivariate analysis. (2) In addition to traditional inflammatory markers, the prognosis of CRC is also influenced by other factors (such as gene mutations, tumor microenvironment, etc.), which may affect the independent value of PLR and NLR.

The following are the shortcomings of this study and suggestions for future research:

1. Larger sample sizes and multicenter studies: As our study had a relatively limited sample size and was a single-center retrospective analysis, future research could expand the sample size through multicenter, prospective studies to validate our findings and increase the generalizability and reliability of the results.
2. Dynamic changes in inflammatory markers: Our study mainly focused on baseline data, while the dynamic changes of inflammatory markers during treatment could have significant prognostic implications. Future studies should investigate the changes in inflammatory markers at different stages of treatment and their predictive value for prognosis.
3. Mechanistic studies: Although our study explored the correlation between inflammatory markers and prognosis and lung metastasis in CRC patients, the specific mechanisms by which these markers influence tumor immune microenvironment and metastasis remain unclear. Future research could utilize experimental models and clinical samples to further elucidate the mechanisms of action of these markers within the tumor microenvironment.
4. External validation: The findings in cohort need to be validated in independent external cohorts to confirm their reliability and reproducibility.

Despite certain limitations, this study demonstrated that inflammatory markers can serve as simple and cost-effective indicators for monitoring postoperative recurrence and metastasis in colorectal cancer (CRC) patients. By applying appropriate statistical methods, we minimized potential biases and controlled for confounding factors, thereby strengthening the reliability of our findings. This will facilitate the screening of patients at high risk of lung metastasis to enhance their follow-up, so as to detect and manage lung metastasis earlier to improve the overall survival of patients.

## Conclusion

We found lower PNI, LMR, and CALLY were significant predictors of reduced progression-free survival (PFS), and PNI and CALLY were independently associated with an increased risk of postoperative lung metastasis. More importantly, the PNI demonstrates high specificity in predicting pulmonary metastasis of colorectal cancer and may serve as a simple and effective peripheral blood biomarker for assessing the risk of ESCC, providing new insights and references for early screening and prevention.

## Data Availability

The datasets used and analysed during the current study are available from the corresponding author on reasonable request.

## Abbreviations

CRC: Colorectal cancer
NLR: Neutrophil-to-lymphocyte ratio
PLR: Platelet-to-lymphocyte ratio
CRP: C-reactive protein
CALLY: CRP–albumin–lymphocyte
PNI: Prognostic nutritional index
LMR: Lymphocyte-to-monocyte ratio
PFS: Progression free survival
SIRI: Systemic immune-inflammation index
CEA: Carcino-embryonic antigen
CXCR: CXC chemokine receptor
CXC: Motif chemokine ligand
TAMs: Tumor-associated macrophages
ROS: Reactive oxygen species
RNS: Reactive nitrogen species
IL-6: Interleukin-6
DNMT1: DNA methyltransferase 1
